# Design and Assessment of a Novel Biconical Human-Sized Alternating Magnetic Field Coil for MNP Hyperthermia Treatment of Deep-Seated Cancer

**DOI:** 10.3390/cancers15061672

**Published:** 2023-03-08

**Authors:** Levan Shoshiashvili, Irma Shamatava, David Kakulia, Fridon Shubitidze

**Affiliations:** 1 Department of Electrical and Electronics Engineering, Faculty of Exact and Natural Sciences, Ivane Javakhishvili Tbilisi State University, 0179 Tbilisi, Georgia; 2 Thayer School of Engineering, Dartmouth College, Hanover, NH 03755, USA

**Keywords:** hyperthermia, MNP, magnetic nanoparticles, cancer, deep-seated tumors, pancreatic cancer, alternating magnetic field, human-sized coil

## Abstract

**Simple Summary:**

A novel human-sized alternating magnetic field (AMF) coil is researched, designed and evaluated using numerical methods to achieve magnetic nanoparticle hyperthermia therapy in deep-seated tumors while avoiding damage to normal tissues. This is achieved by utilizing a circular current’s electric and magnetic field spatial distributions. The studies are done for pancreatic cancer. Computational electromagnetic and temperature distributions are presented for a full-body, 3D human model. The results showed that the proposed human-sized coil could provide clinically relevant AMF to cancerous regions while causing negligible Joule heating to normal tissue, compared to commonly used AMF coils.

**Abstract:**

Magnetic nanoparticle (MNP) hyperthermia therapy is a treatment technique that can be used alone or as an adjunct to radiation and/or chemotherapies for killing cancer cells. During treatment, MNPs absorb a part of electromagnetic field (EMF) energy and generate localized heat when subjected to an alternating magnetic field (AMF). The MNP-absorbed EMF energy, which is characterized by a specific absorption rate (SAR), is directly proportional to AMF frequency and the magnitude of transmitting currents in the coil. Furthermore, the AMF penetrates inside tissue and induces eddy currents in electrically conducting tissues, which are proportional to the electric field (**J** = σ**E**). The eddy currents produce Joule heating (<**J**·**E**> = 0.5·σ·E^2^) in the normal tissue, the rate of energy transfer to the charge carriers from the applied electric fields. This Joule heating contains only the electric field because the magnetic field is always perpendicular to the velocity of the conduction charges, i.e., it does not produce work on moving charge. Like the SAR due to MNP, the electric field produced by the AMF coil is directly proportional to AMF frequency and the magnitude of transmitting currents in the coil. As a result, the Joule heating is directly proportional to the square of the frequency and transmitter current magnitude. Due to the fast decay of magnetic fields from an AMF coil over distance, MNP hyperthermia treatment of deep-seated tumors requires high-magnitude transmitting currents in the coil for clinically achievable MNP distributions in the tumor. This inevitably produces significant Joule heating in the normal tissue and becomes more complicated for a standard MNP hyperthermia approach for deep-seated tumors, such as pancreatic, prostate, liver, lung, ovarian, kidney, and colorectal cancers. This paper presents a novel human-sized AMF coil and MNP hyperthermia system design for safely and effectively treating deep-seated cancers. The proposed design utilizes the spatial distribution of electric and magnetic fields of circular coils. Namely, it first minimizes the SAR due to eddy currents in the normal tissue by moving the conductors away from the tissue (i.e., increasing coils’ radii), and second, it increases the magnetic field at the targeted area (z = 0) due to elevated coils (|z| > 0) by increasing the radius of the elevated coils (|z| > 0). This approach is a promising alternative aimed at overcoming the limitation of standard MNP hyperthermia for deep-seated cancers by taking advantage of the transmitter coil’s electric and magnetic field distributions in the human body for maximizing AMF in tumor regions and avoiding damage to normal tissue. The human-sized coil’s AMF, MNP activation, and eddy current distribution characteristics are investigated for safe and effective treatment of deep-seated tumors using numerical models. Namely, computational results such as AMF, Joule heating SAR, and temperature distributions are presented for a full-body, 3D human model. The SAR and temperature distributions clearly show that the proposed human-sized AMF coil can provide clinically relevant AMF to the region occupied by deep-seated cancers for the application of MNP hyperthermia therapy while causing less Joule heating in the normal tissues than commonly used AMF techniques.

## 1. Introduction

Cancer remains one of the most fatal diseases in the 21st century. According to the American Cancer Society’s estimates, in 2022, there will be approximately 5250 new cancer cases diagnosed each day, which totals about 1,918,030 cases in the year [1]. As a result, about 32% (609,360) of total deaths in the United States are projected to be cancer deaths [2]. Among tumor types, deep-seated cancers, such as lung, pancreatic, prostate, colorectal esophagus, and liver, account for more the 50% (314,020) of cancer deaths in the USA [2]. Pancreatic cancer, particularly pancreatic adenocarcinoma, has the highest mortality rate (about 80%) of all major cancers; for all stages combined, its five-year survival rate is approximately 8% [1,2,3,4]. Currently, systemic chemotherapy and surgical resection are standard treatment options. Recently, the FOLFIRINOX regimen, which combines the drugs leucovorin calcium (folinic acid), fluorouracil, irinotecan hydrochloride, and oxaliplatin [5], has been recommended [6,7] for improving pancreatic cancer patients’ survival rate by 11.1 months. Due to the poor survival of pancreatic cancer patients, over the last several decades, significant efforts have been made to combine chemo and/or radiation therapies with localized heating, such as microwave [8], high-intensity focused ultrasound [9], photodynamic therapy [10], and magnetic nanoparticle hyperthermia (MNPH) [11,12,13] therapies.

Among these heat energy delivery techniques, MNPH therapy, a minimally invasive thermal technique for cancer therapy, has emerged as a potential deep-seated cancer treatment technique. There are several factors that make the MNPH treatment attractive in cancer treatments: first, one must generate high-specificity, localized heat to damage malignant cells, namely an increase in local temperature between 43 and 45 °C is sufficient for cancer cells apoptosis, second different heat-induced damages should be achieved in the tumors and healthy tissues, and finally, the tumor-associated macro-environment should be modified by switching M2 polarized protumor macrophages to M1 polarized antitumor macrophages, which in return produces antitumor immune responses [14]. Due to all these therapeutic benefits of MNPH cancer therapy, MNPH has been evaluated in pre-clinical and clinical settings [11,13,15,16,17,18,19,20]. In general, MNP hyperthermia involves a two-step approach: (1) delivering MNPs to the cancerous tissues/cells and (2) MNP activation using an external AMF. Once MNPs are delivered inside tumor cells, the technology activates electromagnetic fields that transfer energy to the MNPs, resulting in localized heating and tumor cell cytotoxicity [21,22]. Studies have shown hyperthermia to be effective in treating pancreatic cancers. Kossatz et al. [23] have demonstrated the therapeutic effects of MNP hyperthermia on BxPC-3 pancreatic cancer xenografts (human pancreatic adenocarcinoma) in an in vivo murine model. In their study, they injected MNPs locally into the tumor and exposed it to an AMF for 60 min, achieving a temperature of 43 °C, and then conducted histological analyses. The results showed that MNP hyperthermia-treated tumor tissue had decreased cell growth compared to the untreated (i.e., no MNP and no AMP are given) tissue [23]. The applicability of MNP hyperthermia was demonstrated for a murine xenograft model by Basel [24] as well. This study clearly demonstrated that MNP hyperthermia technology could increase the average post-tumor insertion life expectancy by 31% for the murine pancreatic cancer model. In other studies, in vitro and in vivo, Ivkov et al. [13] demonstrated the enhancement of radiation therapy for pancreatic cancer when combined with MNP hyperthermia.

In order to translate these results in mice to a clinical setting, a few major obstacles related to the physics of low-frequency electromagnetic field absorption must be overcome. The rise of local temperature is directly related to the magnitude of the AMF in the tumor, which decays rapidly (as 1/R^2^, where R is the distance from a coil to the tumor) from a coil. As a result, for deeper targets, one would need to increase the transmitter current in the coil to achieve therapeutic magnetic field strength within the tumor. However, high transmitter currents activate not only MNPs in cancerous tissues but also produce a high electric field, **E**, and eddy currents, **J**, within normal tissue that cause non-specific Joule heating (<**J**·**E**>) in the normal tissues [15,25,26]. This significantly limits the applicability of MNP hyperthermia for deep-seated tumors, such as oral melanoma, head-neck, pancreatic, prostate, etc., and imposes limitations on the product of magnetic flux density and frequency (B∙f) for MNP hyperthermia treatment [19,27,28,29,30]. The B∙f limitations that clinical test subjects were able to withstand for more than one hour without major complications have been reported in four independent studies [27,28,29,30]; the limit varies from 562.5 mT·kHz to 6250 mT·kHz) [30]. One way to address this issue is to develop an MNP which produces high SAR at low AMF strength for generating therapeutic temperature within tumors. Another way is to redesign the AMF coil to provide the desired AMF at the tumor while decreasing the Joule heat in the normal tissues as compared to a standard coil setup. Recently, a Dartmouth group has developed flower-like MNPs which exhibit high SAR at low (<20 mT) AMF strength [31]. However, to achieve a therapeutic effect in deep-seated tumors, such as lung, pancreatic, prostate, colorectal esophagus, and liver, it is desirable to develop a new coil for delivering AMF to deep-seated tumors while minimizing undesirable eddy current heating in normal tissues.

This paper introduces a human-sized AMF coil for MNP hyperthermia. The proposed AMF system is a coil with multiple turns of varying radii forming a biconical shape. It takes advantage of the circular coils' electric and magnetic field spatial distributions to minimize eddy currents and maximize AMF at the tumor. The system provides 15 mT magnetic flux density at the tumor for the 133 Ampere alternating transmitter current at 100 kHz frequency, i.e., the *B∙f =* 1500 mT·kHz is well below the upper limit, and keeping the Joule heating temperature below the acceptable level in the normal tissues. The numerical results are given for a full-body, 3D virtual human model to illustrate the applicability of the proposed human-sized coil to AMF application for deep-seated tumors. The SAR and temperature distributions are presented for different size pancreatic cancers and for different MNP distributions to show proof of principle for the new human-sized coil.

## 2. Materials and Methods

This section summarizes numerical methods and MNP that are used in this study. Namely, first, the virtual human (VF) model [32,33] is introduced to assess the applicability of MNPH for deep-seated tumors in humans. Then, the Dartmouth MNP is described, and finally, electric and magnetic field integral equations and bio-heat equations are presented for calculations of electromagnetic fields and temperature distributions inside a VF model subjected to an AMF field produced by a human-sized biconical coil.

### 2.1. Virtual Human Model

**Figure 1:**
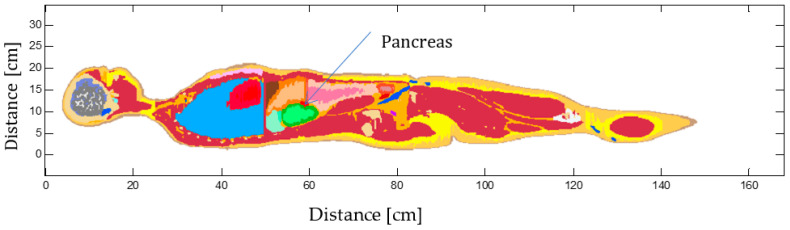
VF-Duke high 2 mm resolution model. The different colors correspond to different tissues.

A virtual human model is used to assess the applicability of the proposed human-size, biconical AMF coil for deep-seated tumors. Specifically, computational studies are done for pancreatic cancer. The electric field, magnetic field, non-specific Joule heating SAR, and temperature distributions are calculated for a virtual family (VF) human model Christ et al. [33]. The VF models consist of four highly detailed anatomically correct whole-body models of an adult male, an adult female, and two children. Figure 1 shows a cross-section of a 2 mm resolution VF-Duke model.

The model consists of up to 84 different tissues and organs. The models are reconstructed as three-dimensional computer-aided design (CAD) objects with high-fidelity anatomical detail. The electromagnetic fields and temperature distributions in the whole body are calculated when the human model is exposed to the AMF produced by the human-sized coil. All subsequent results are presented for the adult male in Figure 1, called Duke in the VF model [33]. The computational domain is divided into Nx · Ny · Nz voxels of size 2 mm × 2 mm × 2 mm, in total 43,538,880 voxels (Nx = 304, Ny = 154, Nz = 930). The tissue electromagnetic and thermal properties, summarized in Table 1, are extracted from a tissue database [34] and assigned to each voxel.

### 2.2. Magnetic Nanoparticles

Although all subsequent studies are done for the Dartmouth gamma-Fe_2_O_3_ MNP, the presented results are applicable to other types of MNP. Dartmouth MNP consists of 2–5 nm crystals in 20–40 nm flower-like aggregates with a mean size of 27 nm and a standard deviation of 5.2 nm. The hydrodynamic diameter has a mean of 110 nm and a standard deviation of 0.33 nm, and the saturation magnetization, remanence, and coercivity are 1.1 emu/g, 0.007 emu/g, and 30 µT (0.3 G), respectively. More detailed information about Dartmouth MNP's shape, size, magnetic properties, and heating mechanism can be found in our previously published manuscripts [31,35,36]. These studies have shown that these particles produce therapeutic levels of SAR at low AMF strength, which makes them advantageous for deep-seated tumor MNP hyperthermia cancer therapy, where high field strengths are not practical using an external coil. For these studies, we selected 15 mT, for which the particles have an SAR of ~55 W/g Fe_2_O_3_ at 100 kHz [31].

### 2.3. Alternating Electromagnetic Fields Calculations

The alternating electromagnetic (**E** and **B**) fields that are produced by the human-sized coil are modeled using the **E** electric and magnetic **B** flux density integral equations, as

(1) $$E=-i\omega A+\frac{1}{i\omega \epsilon \mu }\nabla (\nabla \cdot A)$$

**Figure 2:**
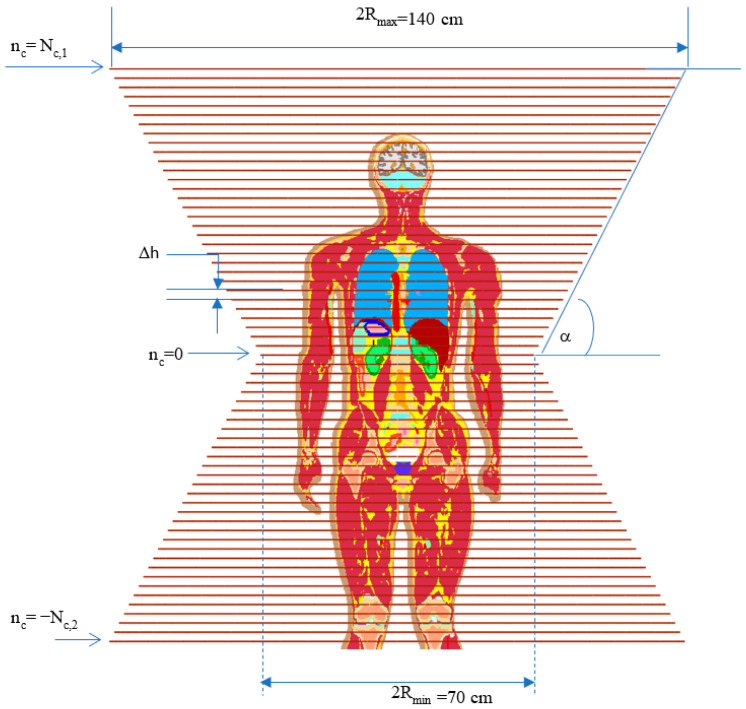
Human-sized coil; The system has a biconical shape and contains 1 + *N_c_*_,1_ + *N_c_*_,2_ turns/coils. Δ*h* is the separation between two nearby coils, $a_{n_{c}}^{}$ = *R*_min_ + Δ*h* (|*n_c_*|) tan(*α*) is the radius of the *n_c_*-th coil, where $n_{c}\in \left[-N_{c,2}:N_{c,1}\right]$. $a_{o}=R_{\min}$ is the radius of the *n_c_* = 0 coil’s radius, and *α* is a half flare angle between the upper and lower cones.

(2) B=∇×A

where in Equations (1) and (2) $A(r)=\frac{\mu }{4\pi }\oint \frac{Ie^{-ikR}}{R}d\ell$ is the magnetic vector potential, *I* is current in the coil, R=|r−r′| is the distance between observation **r** and **r′** source points, dℓ the differential length tangential vector at the **r′** source point, $\mu =\mu_{r}\mu_{o}$, $\varepsilon =\varepsilon_{r}\varepsilon_{o}$, *µ_r_* and $\varepsilon_{r}$ are relative magnetic and electric permeabilities of the medium, respectively, $\mu_{o}=4\pi \cdot 10^{-7}$ [H/m] and εo=1μ0c2 [F/m] are vacuum magnetic permeability and electric permittivity, respectively, c is the speed of light in vacuum, $i=\sqrt{-1}$ is the unit complex number, *ω* is circular frequency, and k is wave number in a medium. For simplicity, the coils are modeled as infinitesimally thin wires. The total AMF (**B**) at the center (*n_c_* = 0) of a biconical coil, Figure 2, carrying *I* current, can be calculated as in [37].

(3) $$B_{z}=\mu \;I_{}\sum_{n_{c}=-N_{c,2}}^{N_{c,1}}\frac{a_{n_{c}}^{2}}{2{\left({\left(\Delta h\cdot n_{c}\right)}_{}^{2}+a_{n_{c}}^{2}\right)}_{}^{\frac{3}{2}}}$$

### 2.4. AMF Coil Design

The most used AMF coils for MNPH consist of a main coil (or pair of coils), which provides a magnetic field, and/or magnetic core, which focuses the AMF over a region of interest (ROI). There are two major issues associated with the design coils, such as, first, the coils should provide a desirable AMF field at the ROI with minimal magnetic field distributions in the surrounding areas, and second, the generation of eddy current (i.e., nonspecific Joule heat) in healthy tissues.

**Figure 3:**
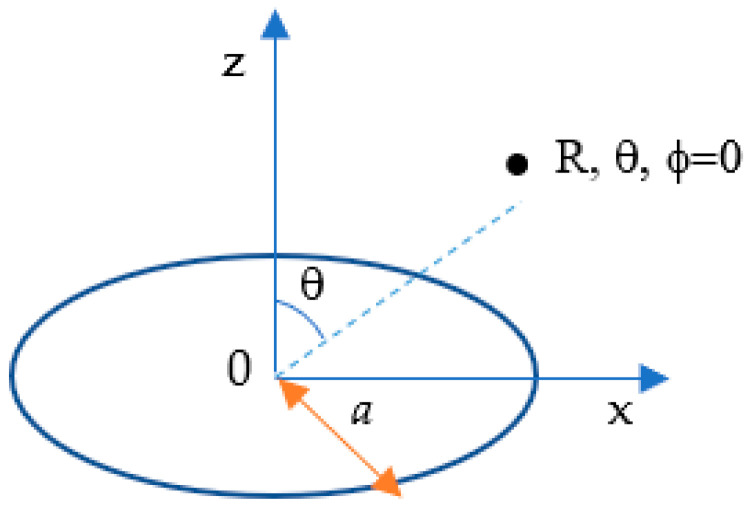
A current-carrying circular coil with *a* radius centered at the origin of a spherical coordinate R, θ, ϕ.

**Figure 4:**
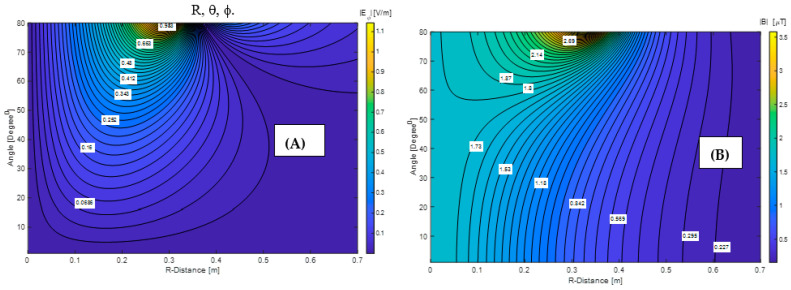
Electric (**A**) and magnetic (**B**) fields distribution for the 35 cm radius circular coil carrying I = 1 A current.

To overcome these issues, an optimal AMF coil geometry was researched using an integral equation solver, which provides the relationship between the current and the resultant electromagnetic field (see Section 2.3). For simplicity, first, the electric and magnetic fields were analyzed for a single circular current coil, Figure 3. Figure 4A,B show electric and magnetic field distributions on the R-θ plane when ϕ = 0 (i.e., y = 0) for the circular coil with a 35 cm radius carrying I = 1 [A] current, respectively. The calculated results show that the magnitude of the electric field decreases as the R-distance decreases. In addition, the electric field peak moves inside the *a* = 35 cm radius coil when R-observation is less than *a.* The magnetic field exhibits a similar but opposite trend, Figure 4. Namely, the magnitude of the magnetic flux density increases as the R-observation point moves toward the center. Using these results, one could achieve the desirable magnetic flux density around the axis of a current-carrying coil with acceptable electric field values by varying the α angle (α = 90 − θ) and the R-observation distance. Based on these results, we have decided to design and investigate a biconical shape coil α = 60° degree half flare angle. The results were evaluated against Helmholtz and solenoidal coils for achieving therapeutic 15 mT AMF at the pancreas. Table 1 Summarizes calculated maximum Joule heat SAR_Joule_ in the VF Duke model and alternating current magnitude during MNPH pancreatic cancer treatment.

Table 2 shows that a single-turn Helmholtz coil will require an impractical ~10 kA current to achieve 15 mT AMF at the pancreas center. The same impractical current value was reported for a single-turn Helmholtz coil by Attaluri et al. in [38]. The comparisons between the 164-turn standard solenoid and biconical coils, operating at 100 kHz, illustrate that the latter coil produces the desirable 15 mT AMF at the pancreas center and the smallest 42 W/kg Joule heating SAR_Joule_ in the normal tissues using the realistic 133 A current. Furthermore, this 42 W/kg Joule heating SAR_Joule_, at 100 kHz AMF and 15 mT magnetic field flux density at the pancreas center, for the proposed biconical coil is much smaller than the SAR_Joule_ = 248 W/kg and SAR_Joule_ = 758 W/kg deposed in a cylindrical shape 0.5 S/m conductive (typical muscle tissue conductivity) homogeneous tissue model placed in the Johns Hopkins University (JHU) Maxwell [38] and in the MagForce MFH300F [39] coils, respectively. The SAR_Joule_ = 248 W/kg and SAR_Joule_ = 758 W/kg are calculated using empirical and analytical expressions provided by Attaluri et al. supplement materials [38].

### 2.5. Bio-Heat Equation

For describing the MNP and non-specific Joule heats transfer in the tissues, Penne’s [40] equation is solved using the finite difference technique [29,41],

(4) $$\rho C\frac{\partial T}{\partial t}=\nabla \left(k\nabla T\right)+Q_{b}+Q_{m}+Q_{joule}+Q_{mnp}$$

where *ρ* (kg/m^3^) is the tissue density, C (J/kg·K) is the heat capacity, *k* (W/(m·K)) is the thermal conductivity, *T* (K) is transient temperature, $Q_{b}$ (W/m^3^), and $Q_{m}$ (W/m^3^) are heat dissipation due to the blood flow and metabolic heat, respectively.

(5) $$Q_{b}=\rho_{b}C_{b}\omega_{b}(T_{a}-T)$$

where $\rho_{b}$ and $C_{b}$ are blood density and blood heat capacity, $T_{a}$ (°C) and T (°C) are arterial blood and tissue temperature, respectively, and $\omega_{b}$ (m^3^/s/kg) is the heat transfer rate in blood. These parameters are summarized in Table 1.

In Equation (4), the term $Q_{joule}$ is non-specific Joule heat (<j·E> = 0.5σE^2^) due to the E electric field in the σ conducting tissue, and $Q_{mnp}$ is heat produced by the MNP in the tumor when subjected to an external AMF. The thermophysical and electromagnetic properties of the tissue are extracted from a tissue database [34] and summarized in Table 1. In all subsequent calculations, the convection boundary conditions are set between skin-air surface with the convection coefficient $=10\;W/m^{2}$.

## 3. Results

In this section, we present electromagnetic fields, SAR, and temperature simulation results for the VF Duke model. All subsequent calculations are done for pancreatic cancer. First, AMF and Joule heating SAR_Joule_ distributions are illustrated for the high (2 mm) resolution Duke model subjected to alternating electromagnetic (AEMF) produced by a single-turn Helmholtz coil. Then the temperature and Joule heating SAR_Joule_ distributions are presented for the same VF-Duke model placed in AEMF generated by the novel biconical coil. Finally, the applicability of the combined human-sized biconical coil and MNPH is assessed for the treatment of deep-seated tumors.

### 3.1. Analysis of the Limitations of the Standard Approach

**Figure 5:**
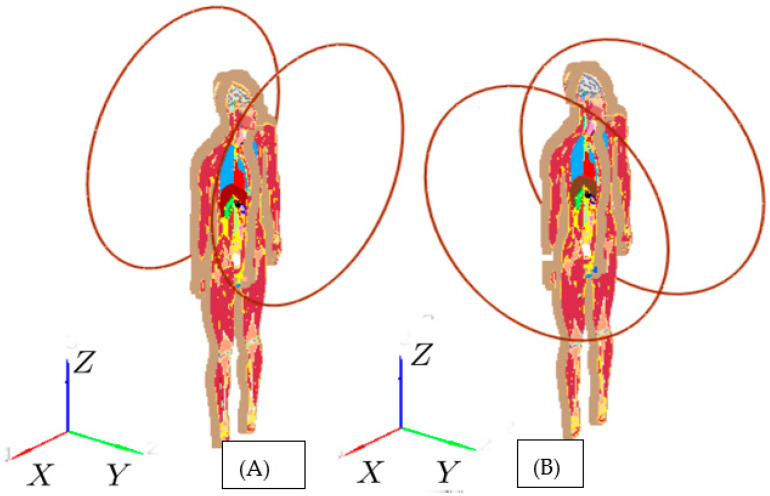
Helmholtz coils are placed: (**A**) front and back; (**B**) left and right. The coils are centered with respect to the pancreas’ center (x = 28.6 cm, y = 17.2 cm, z = 123.6 cm).

**Figure 6:**
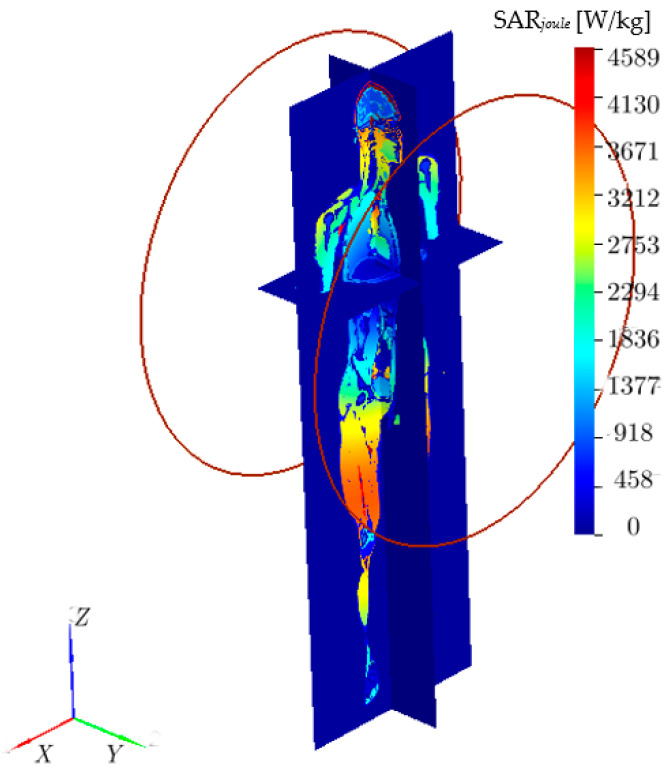
The Joule heating SAR*_Joule_* [W/kg] distribution inside the VF Duke model.

To illustrate the limitations of the standard approach for deep-seated tumors, we conducted numerical calculations of AMF distributions inside the 2 mm-resolution VF Duke model (Figure 1) at 100 kHz. The AEMFs are produced by 60 cm diameter Helmholtz coils in three configurations. In the first configuration Figure 5A), coils are placed front (centered at x = 28.6 cm, y = 47.2 cm, z = 123.6 cm) and back (centered at x = 28.6 cm, y = −13.8 cm, z = 123.6 cm), and in the second configuration Figure 5B), coils are placed left (centered at x = −1.4 cm, y = 17.2 cm, z = 123.6 cm) and back (centered at x = 58.6 cm, y = 17.2 cm, z = 123.6 cm). The coils centers are aligned to the pancreas center (x = 28.6 cm, y = 17.2 cm, z = 123.6 cm). Studies in [31] showed that for achieving therapeutic levels of heating at the pancreas, the Dartmouth MNPs, which provide high SAR due to MNP at low AMF strength [31], would require at least 15 mT. Figure 6 shows the Joule heating SAR*_Joule_* distributions inside the VF Duke model. The 10K ampere currents are required in the single-turn coils to achieve the required 15 mT magnetic field at the pancreas’ center. The maximum SAR*_Joule_* = 22.5 KW/kg is observed in spinal cerebrospinal fluid. This high SAR*_Joule_* resulted in a temperature rise of 100-s °C in the normal tissues in less than 1 min. Similar results were observed for the Helmholtz coil placed symmetrically to the *x*-axis ((A) and (B), see Figure 5) and *z*-axis.

Overall, these simulations clearly show that the standard approaches, which use single (or multi closely spaced) turn coils, are not applicable for deep-seated tumors, and an alternative approach that can supply sufficient field strength at the tumor with tolerable eddy current heating is needed.

### 3.2. Human-Sized Biconical Coil: SAR_Joule_ and Temperature Distributions

A series of electromagnetic and bio-heat-temperature calculations were conducted for human-sized biconical-shaped coils using the electromagnetic volume integral and bio-heat equations solvers developed by our group and validated against experimental and analytical data [29,31,41]. Here, the main goal was to determine the human coil’s optimal size and number of turns that will provide at least a 15 mT magnetic field at the pancreas for achieving therapeutic levels of MNP heating in the tumor while minimizing the Joule heating in the normal tissue. The simulations were done for the human-sized coil at 100 kHz frequency. Attention was given to temperature and SAR_Joule_ distributions in and around the spine and the brain for two reasons. First, the spinal cord contains the most electrically conductive human tissue in the body and lacks significant temperature regulation, see Table 1. Second, although the brain has better temperature regulation but is highly sensitive to temperature elevation, it is reported that at temperatures between 42 and 43 °C, neurons can be damaged permanently [42]. After a series of calculations, the human-sized biconical coil that provides the desired AMF and SAR_Joule_ distributions was determined to be a double-layer biconical coil, with the inner and outer layers radius of *R*_min_ = 35 cm and *R*_min_ = 37.5 cm, respectively. The separation between nearby coils was set to be Δ*h* = 2.5 cm, and the half flare angle α = 60°; between upper and low cones was determined to be α = 60° for both coils. The number of turns was chosen to be *N_c_*_,1_ = 30; *N_c_*_,2_ = 51. Note that the Δ*h* = 2.5 cm is chosen to use for constructing a realistic size current carrying tube.

**Figure 7:**
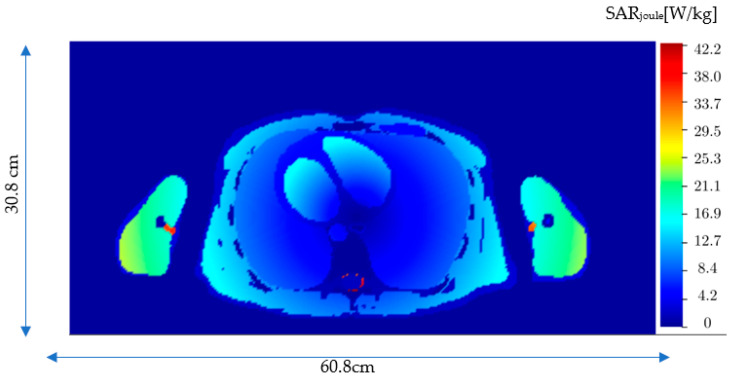
The Joule heating specific absorption rate (SAR_Joule_) distribution in the xy plane (z = 108.6 mm) where SAR_Joule_ is observed in the VF Duke model.

**Figure 8:**
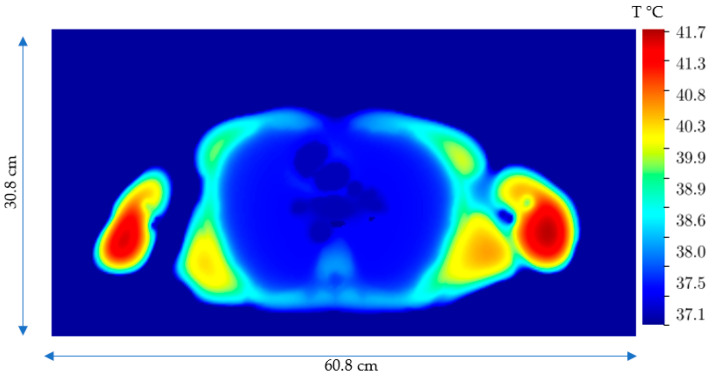
Temperature distribution after 20 min on the xy plane (z = 140.6) with the maximum temperature due to the Joule heating, SAR_Joule_.

The steady-state temperature was calculated for the VF Duke model by dividing the entire computation volume into Nx · Ny · Nz = 43,538,880 voxels of size 2 mm × 2 mm × 2 (Nx = 304, Ny = 154, Nz = 930). We assumed that the VF Duke model was placed at room temperature, 22 °C. The simulations were run for 8 h to reach steady-state conditions to establish the baseline. The steady-state was then used as an initial temperature distribution to calculate the induced temperature due to Joule heating and MNP heading. Figure 7 shows the Joule heating, SAR_Joule_, distribution on a plane containing the maximum SAR_Joule_. As expected, the maximum SAR_Joule_ is in the cerebrospinal fluid within the spine. Figure 7 shows the temperature distribution after 20 min in the same plane containing the maximum SAR_Joule_. The result shows that although the maximum SAR_Joule_ is within the cerebrospinal fluid, the maximum temperature after 20 min is registered in arms, which are close to the coils. The temperature distribution after 20 min on the plane with maximum temperature due to the Joule heating, SAR_Joule_, is depicted in Figure 8.

The maximum induced Joule heating temperature reaches about 41.7 °C in the shoulder regions, which are close again to the coils. Although the temperature elevation in the arms and shoulders is below 42 °C, one could utilize other techniques, such as repositioning the patient’s arms or using surface cooling pads to further manage this temperature.

**Figure 9:**
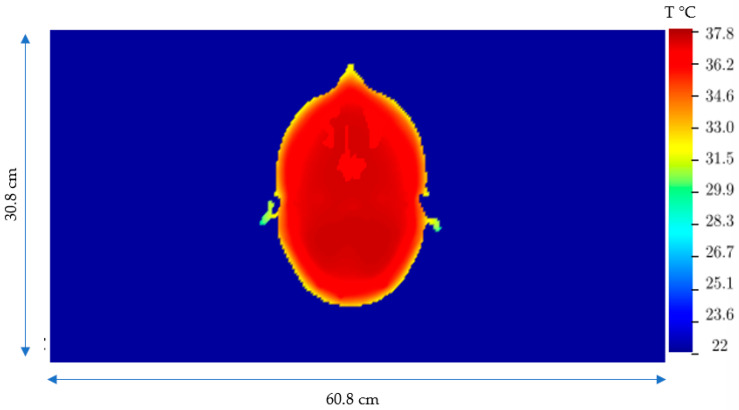
Steady-state temperature distribution in the brain in the plane with the maximum T due to the Joule heating, SARJoule.

**Figure 10:**
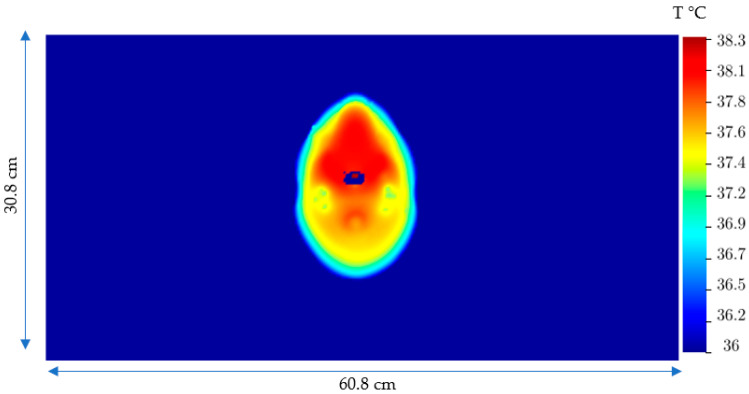
2D Temperature distribution after 20 min in the brain in the plane with maximum T due to the Joule heating, SARJoule.

Furthermore, we calculated and analyzed the steady state and the Joule heat-induced temperature in the brain. The calculated temperatures are depicted in Figure 9 and Figure 10 in the plane with the maximum T due to the Joule heating, SAR_Joule_. The studies illustrate that a maximum temperature rise (see Figure 9 and Figure 10) is less than 1 °C in the brain after 20 min MNPH treatment. This is significantly below 42 °C, the acceptable maximum brain temperature for avoiding permanent damage to neurons [42].

### 3.3. Human-Sized Coil: Assessing MNPH Efficacy and Temperature Distributions

**Figure 11:**
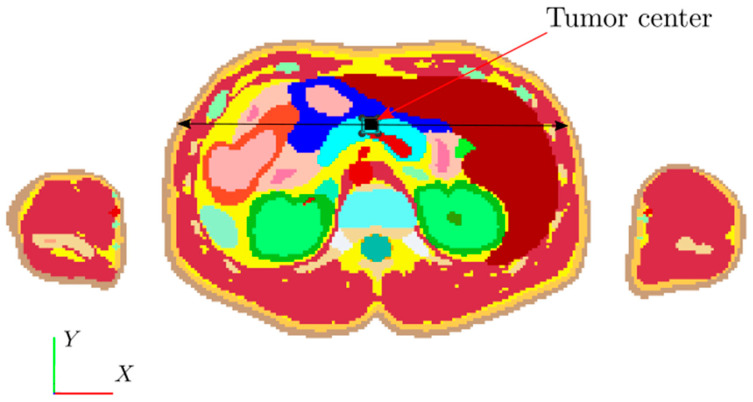
2D slice of 3D VF model at pancreas plane. The black square corresponds to a 1 cm^3^ tumor in the xy cross-section at z = 123.6 cm. In the lower-left corner, 1, 2, and 3 are the *x*, *y*, and *z* axes. The black double arrow line is the observation line.

Finally, results are given to demonstrate the applicability of the human-sized coil for MNPH therapy. Although all subsequent calculations are done for the flowerlike Dartmouth MNP with a concentration of 63.5 mgFe_2_O_3_/mL in 1 mL water, these results are applicable to other types of MNPs as well. We assume that Dartmouth MNP is delivered in the tumor via local injection and activated with the human-sized biconical AEMF coil operating at a frequency of 100 kHz and providing a 15 mT magnetic field at the tumor. The SAR_mnp_ = 55 W/g Fe_2_O_3_ due to MNP was estimated from [31]. Three 1 cm^3^, 2 cm^3^, and 3 cm^3^ size tumors are considered. Tumors are modeled as a rectangular parallelepiped. Studies are demonstrated for each size tumor receiving the equivalent of three 3 µL MNP per gram tumor, 5 µL MNP per gram tumor, and 10 µL MNP per gram tumor dose. Figure 11 shows the cross-section of the xy plane at the z = 123.6 slice containing the 1 cm^3^ cubic shape tumor (black).

Full 3D-EM and bio-heat equations are solved for the VF Duke model, subjected to AEMFs produced by the human-sized coil. (A) and (B) graphs show modeled results for 3 µL and 5 µL Dartmouth MNP concentration per gram tumor, respectively. The results are shown at different observation times of AMF exposure.

**Figure 12:**
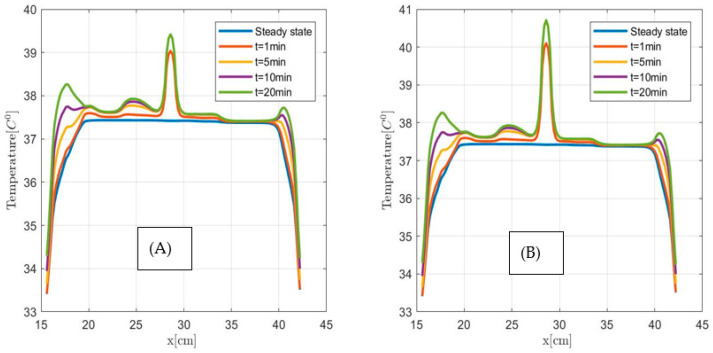
Temperature versus distance along the observation line (the black double arrow line in Figure 11) for the 1 cm^3^ size pancreatic tumor. (**A**) for 3 µL and (**B**) 5 µL Dartmouth MNP concentration per gram tumor.

**Figure 13:**
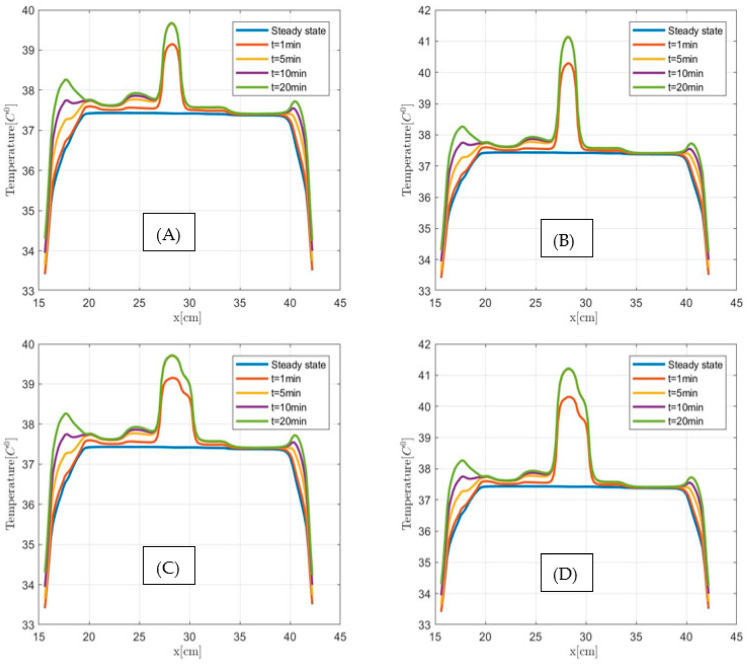
Temperature versus distance along the observation line (the black double arrow line see Figure 11) for 2 cm^3^ (**A**,**B**) and 3 cm^3^ (**C**,**D**) size pancreatic tumors at steady state and after 1 min, 5 min, 10 min and 20 min MNPH exposure times. (**A**,**C**) graphs show modeled results for 3 µL and (**B**,**D**) figures for 5 µL Dartmouth MNP concentration per gram tumor, respectively.

The 2 cm^3^ tumor was modeled by adding 1 cm^3^ volume to the 1 cm^3^ cubic tumor (Figure 11), and the 3 cm^3^ was modeled by adding 1 cm^3^ volume above the 2 cm^3^ tumor model along the *z*-axis. We assumed that the modeled tumors and healthy pancreases have the same mass density and thermal properties, see Table 1. Figure 12 and Figure 13 show the calculated temperature versus distance for 1 cm^3^, 2 cm^3^ and 3 cm^3^ size pancreatic tumors before (steady state) and during MNPH treatment at times t = 1 min, 5 min, 10 min, and 20 min. The MNPH-induced temperature distributions are calculated for the human size biconical coil running 100 kHz and 134 A alternating current and producing a 15 mT field at the pancreas. Figure 12A,B and Figure 13A–D correspond to temperature distributions at different times for 1 µL, 3 µL and 5 µL Dartmouth MNP concentration per gram tumor, respectively. The results show that MNPH-induced temperature is localized in the area containing MNP and rises above the steady-state temperature and Joule heating-induced temperature levels in the normal tissues. Even though the MNPH-induced temperatures do not exceed 42 °C to achieve tumor cell apoptosis, the predicated temperature change could be used for localized drug release and mild hyperthermia.

**Figure 14:**
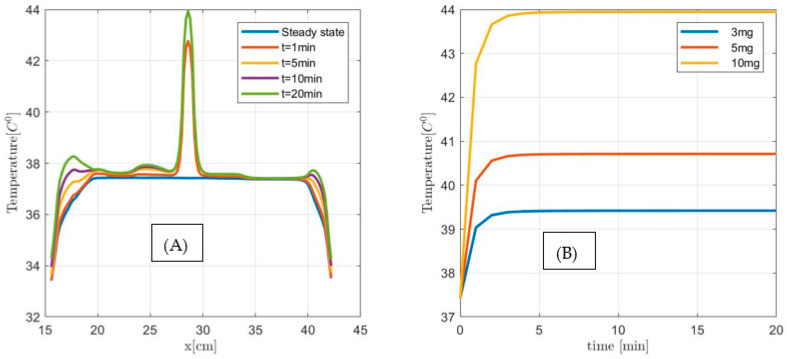
(**A**) Temperature versus distance along the observation line (the black double arrow line see Figure 11) for 1 cm^3^ size pancreatic tumor with 10 µL MNP for steady state, and after 1 min, 5 min, and 10 min exposure time. (**B**) Temperature versus time at the center of 1 cm^3^ size pancreatic tumor for 3 µL (3 mg), 5 µL (5 mg), and 10 µL (10 g) MNP per gram tumor.

**Figure 15:**
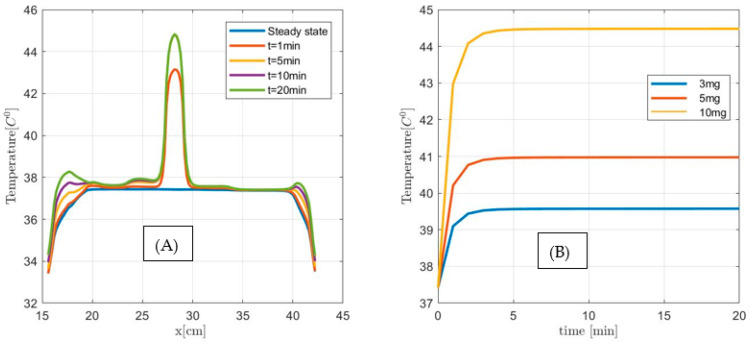
(**A**) Temperature versus distance along the observation line (the black double arrow line see Figure 11) for a 2 cm^3^ size pancreatic tumor with 10 µL MNP for steady state, and after 1 min, 5 min, and 10 min exposure time. (**B**) Temperature versus time at the center of 1 cm^3^ size pancreatic tumor for 3 µL (3 mg), 5 µL (5 mg), and 10 µL (10 g) MNP per gram tumor.

**Figure 16:**
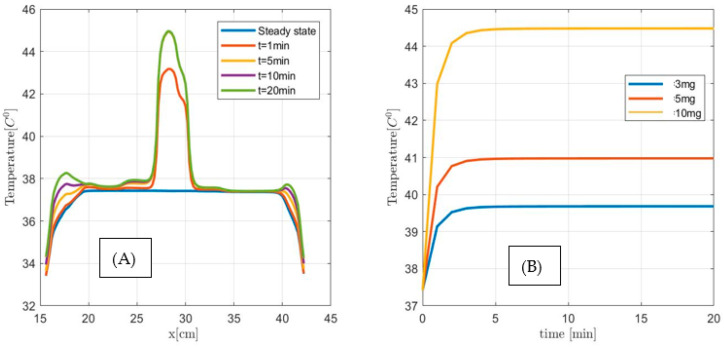
(**A**) Temperature versus distance along the observation line (the black double arrow line see Figure 11) for a 3 cm^3^ size pancreatic tumor with 10 µL MNP for steady state, and after 1 min, 5 min, and 10 min exposure time. (**B**) Temperature versus time at the center of 1 cm^3^ size pancreatic tumor for 3 µL (3 mg), 5 µL (5 mg), and 10 µL (10 g) MNP per gram tumor.

Next, we investigate the applicability of the MNPH using 10 µL Dartmouth MNP per gram tumor concentration for the same size (1 cm^3^, 2 cm^3^, and 3 cm^3^) pancreatic tumor. Figure 14, Figure 15 and Figure 16 show the temperature distributions in each size of pancreatic tumor before and during MNPH. Figure 14A, Figure 15A and Figure 16A display the temperature distribution along the observation line, and Figure 14B, Figure 15B and Figure 16B graphs show the temperature versus time at the center of each 1 cm^3^, 2 cm^3^, and 3 cm^3^ in size tumor, respectively. These results demonstrate that 10 µL Dartmouth MNP per gram tumor concentration can provide therapeutic temperatures for all three differently-sized tumors.

## 4. Discussion

A new human-sized biconical shape coil in combination with Dartmouth magnetic nanoparticles (MNPs) is presented and assessed as a viable approach for deep-seated cancer treatment. Numerical results using a 3D electromagnetic and bio-heat equations solver for a high-resolution virtual human model (VF Duke model) indicate that this approach can effectively produce therapeutic temperatures in pancreatic tumors while minimizing temperature rises in healthy tissues. This is achieved by taking advantage of the circular coil’s electric and magnetic fields’ spatial distributions. Namely, on the one hand, it minimizes magnitudes of the induced eddy currents in healthy tissues by increasing the coils’ radii, and on the other hand, it increases the AMF due to nearby coils (|z| > 0) at the targeted area (z = 0) by increasing off (|z| > 0) coils’ radii gradually. Overall, this decreases the electric field and non-specific Joule heating in the human body while maintaining a consistent magnetic field at the targeted area of the pancreas. As a result, our calculations show that the proposed new human-sized biconical produces much smaller eddy current SAR-s in healthy tissues than the Johns Hopkins University (JHU) Maxwell [38] and in the MagForce MFH300F [39] coils.

**Figure 17:**
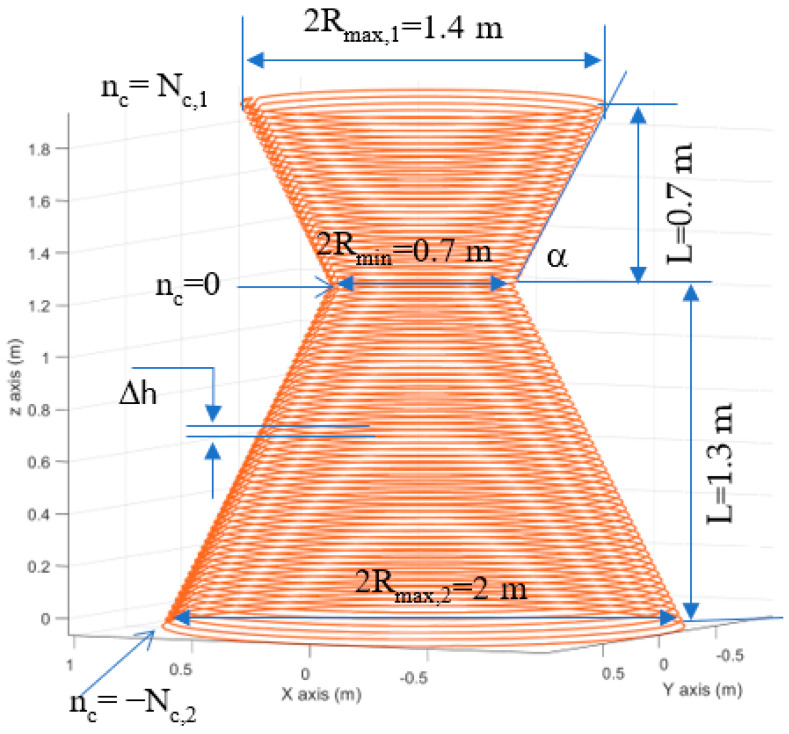
The modeled human-sized coil made of 1cm diameter copper wire, α = 60°, *N_c_*_,1_ =30, *N_c_*_,2_= 54, Δ*h* = 0.025 m.

One way to realize a biconical coil is to build an LC resonant circuit using the biconical coil and a matching capacitor. At the resonant frequency $f=1/\left(2\pi \sqrt{LC}\right),$ the reactance of the inductor and the capacitor cancel each other out in the LC circuit, allowing the maximum current flow through the circuit. Constructing an LC circuit at the resonant *f* = 100 kHz frequency using the biconical coil requires a matching C capacitor with the desired capacitance value. To determine the capacitance, we modeled the human-sized biconical coil, Figure 17, connected to a capacitor and power source in series using the full 3D Maxwell equation solver software package called EMCoS studio [43].

**Figure 18:**
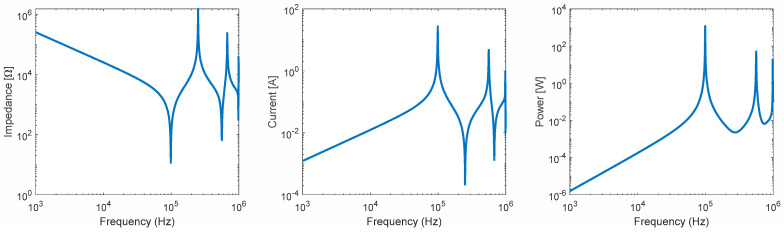
Impedance, current, and power vs. frequency for a biconical coil.

After performing a set of calculations, the required capacitance value for the resonant *f* = 100 kHz frequency LC circuit was determined to be C = 0.625 nF. Figure 18 shows the calculated impedance (Ohm), current (A), and power(W) versus Frequency for the biconical coil connected to the external V = 312.5 V voltage source and C = 0.625 nF capacitor in series. These results show that the human-sized biconical coil can generate a desirable 133 A current and deliver 25 kW of power at a 100 kHz frequency.

Ultimately, the greatest limitation of this technique will likely be the MNP biodistributions in non-cancerous tissues. Such as Prijic et al. [44] have reported that the majority of the intravenously injected, systemically delivered MNP is accumulated in the liver, spleen, and kidneys. Since these vital organs are close to the pancreas, they will be placed in the alternating magnetic field. Consequently, MNPs will produce undesirable heat in the liver, spleen, and kidneys. This lack of specificity of AMF distribution can limit treatment efficacy by limiting the maximum safe applied field strength, thus limiting MNP heating of the pancreatic tumor. One of the ways to overcome this limitation is to place a passive ferrite material, such as a low SAR ferrofluid or flexible ferrite material, next to the targeted tumor to focus and/or direct the alternating magnetic field. In addition, the proposed human-sized coil was not optimized for systemically delivered MNP; hence, these results warrant further study of human-sized coil design in combination with a magnetic field focusing and targeting approach when applied to specific deep-seated tumors with complex tissue geometry.

## 5. Conclusions

The novel biconical human-sized coil delivers the desirable 15 mT AMF at pancreatic cancer at a smaller alternating current (~133 A) than the Helmholtz (~10 KA current) and solenoidal (156 A) coils. The coil induces minimum non-specific Joule heating of the normal tissues and achieves the therapeutic temperature (>42 °C) level in tumors containing 10 µL Dartmouth MNPs per gram tumor concentration by utilizing the circular coil’s electric and magnetic fields spatial distributions. It improves upon the performance of a simple Helmholtz, JHU Maxwell and the MagForce MFH300F by providing clinically acceptable non-specific temperature distributions in the human body while creating the desirable AMF at the target area. Overall, the presented numerical results illustrate an innovative way to deliver AMF and activate MNPs at a previously unachievable distance with clinically viable levels of Joule heating, potentially opening new opportunities to extend the use of MNPH to deep-seated cancers. As a next step before bringing the system into clinical settings, we plan to build a prototype system and conduct MNPH studies on large animals.

## Figures and Tables

**Table 1:** Tissue EM and Thermal parameters.

Material	Electric Conductivity σ (S/m)	Density ρ (kg/m^3^)	Heat Capacity (J/kg/°C)	Thermal Conductivity (W/m/°C)	Heat Generation Rate (W/kg)	Heat Transfer Rate 1.667 × 10^−8^ (m^3^/s/kg)
Air	0	1	1000	0	0	0
Adrenal gland	0.620009	1028	3513	0.44	22.58	1458
Air internal	0	1	1004	0	0	0
Artery	0.705077	1102	3617	0.52	0	10,000
Bladder	0.219954	1086	3581	0.52	0	78
Blood vessel	0.705077	1060	3306	0.52	0	10,000
Bone	0.02089	1908	1313	0.32	0.15	10
Brain grey matter	0.136564	1045	3696	0.55	15.54	764
Brain white matter	0.083885	1041	3583	0.48	4.32	212
Bronchi	0.341515	1102	3306	0.46	3.69	238
Bronchi_lumen	0	1	1004	0	0	0
Cartilage	0.179826	1100	3568	0.49	0.54	35
Cerebellum	0.15662	1045	3653	0.51	15.67	770
Cerebrospinal fluid	2	1007	4096	0.57	0	0
Commissura anterior	0.083885	1041	3583	0.48	4.32	212
Commissura posterior	0.083885	1041	3583	0.48	4.32	212
Connective tissue	0.388886	1027	2372	0.39	0.58	37
Cornea	0.508027	1062	3615	0.54	0	38
Diaphragm	0.369328	1090	3421	0.49	2.44	99
Ear cartilage	0.179826	1100	3568	0.49	0.54	35
Ear skin	0.000628	1109	3391	0.37	1.65	106
Epididymis	0.441861	1082	3778	0.52	3.09	200
Esophagus	0.537457	1040	3500	0.53	2.94	190
Esophagus lumen	0	1	1004	0	0	0
Eye lens	0.200834	1076	3133	0.43	0	0
Eye Sclera	0.521255	1032	4200	0.58	5.89	380
Eye vitreous humor	1.50003	1005	4047	0.59	0	0
Fat	0.043458	911	2348	0.21	0.51	33
Gallbladder	0.900145	1071	3716	0.52	0.46	30
Heart lumen	0.705077	1050	3617	0.52	0	10,000
Heart muscle	0.224545	1081	3686	0.56	39.45	1026
Hippocampus	0.136564	1045	3696	0.55	15.54	764
Hypophysis	0.539026	1053	3687	0.51	13.71	885
Hypothalamus	0.136564	1045	3696	0.55	15.54	764
Intervertebral disc	0.830007	1100	3568	0.49	0.54	35
Kidney cortex	0.177821	1049	3587	0.53	18.43	3874
Kidney medulla	0.177821	1044	3745	0.54	2.85	599
Large intestine	0.25011	1088	3655	0.54	11.85	765
Large intestine lumen	0.369328	1045	3801	0.56	0	0
Larynx	0.179826	1100	3568	0.49	0.54	35
Liver	0.092243	1079	3540	0.52	9.93	860
Lung	0.109608	394	3886	0.39	6.21	401
Mandible	0.02089	1908	1313	0.32	0.15	10
Marrow red	0.10297	1029	2666	0.28	2.09	135
Medulla oblongata	0.15662	1046	3630	0.51	11.37	559
Meniscus	0.179826	1100	3568	0.49	0	35
Midbrain	0.15662	1046	3630	0.51	11.37	559
Mucosa	0.369328	1102	3150	0.34	9.19	594
Muscle	0.369328	1090	3421	0.49	0.91	37
Nerve	0.085742	1075	3613	0.49	2.48	160
Pancreas	0.539026	1087	3164	0.51	11.89	767
Patella	0.02089	1908	1313	0.32	0.15	10
Penis	0.319555	1102	3306	0.46	0.19	12
Pharynx	0	1	1004	0	0	0
Pineal body	0.539026	1053	3687	0.51	13.71	885
Pons	0.15662	1046	3630	0.51	11.37	559
Prostate	0.441861	1045	3760	0.51	6.1	394
SAT	0.043458	911	2348	0.21	0.51	33
Skin	0.000628	1109	3391	0.37	1.65	106
Skull	0.02089	1908	1313	0.32	0.15	10
Small intestine	0.603279	1030	3595	0.49	15.89	1026
Small intestine lumen	0.369328	1045	3801	0.56	0	0
Spinal cord	0.085742	1075	3630	0.51	2.48	160
Spleen	0.124573	1089	3596	0.53	24.11	1557
Stomach	0.537457	1088	3690	0.53	7.13	460
Stomach lumen	0.369328	1045	3801	0.56	0	0
Teeth	0.02089	2180	1255	0.59	0	0
Tendon ligament	0.388886	1142	3432	0.47	0.45	29
Testis	0.441861	1082	3778	0.52	3.09	200
Thalamus	0.136564	1045	3696	0.55	13.93	685
Thymus	0.630011	1023	3043	0.34	3.83	247
Thyroid gland	0.539026	1050	3609	0.52	87.1	5634
Tongue	0.290727	1090	3421	0.49	1.21	78
Trachea	0.341515	1080	3568	0.49	0.54	35
Trachea lumen	0	1	1004	0	0	0
Ureter urethra	0.319555	1102	3306	0.46	2.91	188
Vein	0.705077	1050	3617	0.52	0	10,000
Vertebrae	0.02089	1908	1313	0.32	0.15	10
Tumor	0.539026	1087	3164	0.51	11.89	767

**Table 2:** Coils’ type, size, number of turns, max SAR and current.

Coil Type	*a* Radius [cm]				*N* (Number of Turns)	SAR*_JouleMax_* [W/kg]	*I* [A]
Helmholtz symmetric respect *x*-axis	60				1	22,582	10,000
Helmholtz symmetric respect *z*-axis	60				1	5773	9900
	Inner layer		Outer layer				
Double layer solenoid	*a*_min_ = 35		*a*_max_ = 37.5		164	70	156
Double layer biconical	*a*_min_ = 35	*a*_max_ = 70	*a*_min_ = 37.5	*a*_max_ = 72.5	164	42	133

## Data Availability

The data supporting this study’s findings are available on request from the corresponding author.

## References

[1] Stauffer P.R., Cetas T.C., Jones R.C. (1984). Magnetic Induction Heating of Ferromagnetic Implants for Inducing Localized Hyperthermia in Deep-Seated Tumors. IEEE Trans. Biomed. Eng..

[2] Siegel R.L., Miller K.D., Fuchs H.E., Jemal A. (2022). Cancer statistics, 2022. CA Cancer J. Clin..

[3] Ruarus A., Vroomen L., Puijk R., Scheffer H., Meijerink M. (2018). Locally Advanced Pancreatic Cancer: A Review of Local Ablative Therapies. Cancers.

[4] Kamisawa T., Wood L.D., Itoi T., Takaori K. (2016). Pancreatic cancer. Lancet.

[5] Conroy T., Desseigne F., Ychou M., Bouche O., Guimbaud R., Becouarn Y., Adenis A., Raoul J.L., Gourgou-Bourgade S., de la Fouchardiere C. (2011). FOLFIRINOX versus gemcitabine for metastatic pancreatic cancer. N. Engl. J. Med..

[6] Ducreux M., Cuhna A.S., Caramella C., Hollebecque A., Burtin P., Goere D., Seufferlein T., Haustermans K., Van Laethem J.L., Conroy T. (2015). Cancer of the pancreas: ESMO Clinical Practice Guidelines for diagnosis, treatment and follow-up. Ann. Oncol..

[7] Khorana A.A., Mangu P.B., Berlin J., Engebretson A., Hong T.S., Maitra A., Mohile S.G., Mumber M., Schulick R., Shapiro M. (2017). Potentially Curable Pancreatic Cancer: American Society of Clinical Oncology Clinical Practice Guideline Update. J. Clin. Oncol..

[8] Vogl T.J., Panahi B., Albrecht M.H., Naguib N.N.N., Nour-Eldin N.A., Gruber-Rouh T., Thompson Z.M., Basten L.M. (2018). Microwave ablation of pancreatic tumors. Minim. Invasive Ther. Allied Technol..

[9] Lafond M., Lambin T., Drainville R.A., Dupré A., Pioche M., Melodelima D., Lafon C. (2022). Pancreatic Ductal Adenocarcinoma: Current and Emerging Therapeutic Uses of Focused Ultrasound. Cancers.

[10] Huggett M.T., Jermyn M., Gillams A., Illing R., Mosse S., Novelli M., Kent E., Bown S.G., Hasan T., Pogue B.W. (2014). Phase I/II Study of Verteporfin Photodynamic Therapy in Locally Advanced Pancreatic Cancer. Br. J. Cancer.

[11] Attaluri A., Kandala S.K., Zhou H., Wabler M., DeWeese T.L., Ivkov R. (2020). Magnetic nanoparticle hyperthermia for treating locally advanced unresectable and borderline resectable pancreatic cancers: The role of tumor size and eddy-current heating. Int. J. Hyperth..

[12] Kucharczyk K., Kaczmarek K., Jozefczak A., Slachcinski M., Mackiewicz A., Dams-Kozlowska H. (2021). Hyperthermia treatment of cancer cells by the application of targeted silk/iron oxide composite spheres. Mater. Sci. Eng. C.

[13] Ivkov R., Attaluri A., Guiriba T., Liu Y., Zhou H., Hedayati M., DeWeese T., Liapi E., Herman J. (2014). Magnetic Nanoparticle Hyperthermia and Radiation for Locally Advanced Pancreas Cancer: An In Vitro and In Vivo Study. Int. J. Radiat. Oncol. Biol. Phys..

[14] Deng X., Liang H., Yang W., Shao Z. (2020). Polarization and function of tumor-associated macrophages mediate graphene oxide-induced photothermal cancer therapy. J. Photochem. Photobiol. B Biol..

[15] Wust P., Gneveckow U., Johannsen M., Nohmer D., Henkel T., Kahmann F., Sehouli J., Felix R., Ricke J., Jordan A. (2006). Magnetic nanoparticles for interstitial thermotherapy—Feasibility, tolerance and achieved temperatures. Int. J. Hyperth..

[16] Mendez M.H., Joh D.Y., Gupta R., Polascik T.J. (2015). Current Trends and New Frontiers in Focal Therapy for Localized Prostate Cancer. Curr. Urol. Rep..

[17] Maier-Hauff K., Rothe R., Scholz R., Gneveckow U., Wust P., Thiesen B., Feussner A., Von Deimling A., Waldoefner N., Felix R. (2007). Intracranial thermotherapy using magnetic nanoparticles combined with external beam radiothera-py: Results of a feasibility study on patients with glioblastoma multiforme. J. Neurooncol..

[18] Espinosa A., Kolosnjaj-Tabi J., Abou-Hassan A., Sangnier A.P., Curcio A., Silva A.K.A., Di Corato R., Neveu S., Pellegrino T., Liz-Marzán L.M. (2018). Magnetic (Hyper)Thermia or Photothermia? Progressive Comparison of Iron Oxide and Gold Nanoparticles Heating in Water, in Cells, and In Vivo. Adv. Funct. Mater..

[19] Johannsen M., Gneveckow U., Taymoorian K., Thiesen B., Waldöfner N., Scholz R., Jung K., Jordan A., Wust P., Loening S.A. (2007). Morbidity and quality of life during thermotherapy using magnetic nanoparticles in locally recurrent prostate cancer: Results of a prospective phase I trial. Int. J. Hyperth..

[20] Clinical Trials: NCT02033447 (Prostate), NCT00848042 (Head and Neck Tumors). NCT02033447.

[21] Iacovita C., Florea A., Scorus L., Pall E., Dudric R., Moldovan A.I., Stiufiuc R., Tetean R., Lucaciu C.M. (2019). Hyperthermia, Cytotoxicity, and Cellular Uptake Properties of Manganese and Zinc Ferrite Magnetic Nanoparticles Synthesized by a Polyol-Mediated Process. Nanomaterials.

[22] Ito A., Kuga Y., Honda H., Kikkawa H., Horiuchi A., Watanabe Y., Kobayashi T. (2004). Magnetite nanoparticle-loaded anti-HER2 immunoliposomes for combination of antibody therapy with hyperthermia. Cancer Lett..

[23] Kossatz S., Ludwig R., Dähring H., Ettelt V., Rimkus G., Marciello M., Salas G., Patel V., Teran F.J., Hilger I. (2014). High Therapeutic Efficiency of Magnetic Hyperthermia in Xenograft Models Achieved with Moderate Temperature Dosages in the Tumor Area. Pharm. Res..

[24] Basel M., Balivada S., Wang H., Shrestha T.B., Seo G.M., Pyle M., Ayabaweera G., Dani R.K., Koper O.B., Tamura M. (2012). Cell-delivered magnetic nanoparticles caused hyperthermia-mediated increased survival in a murine pancreatic cancer model. Int. J. Nanomed..

[25] Brezovich A.I., Atkinson W.J., Lilly M.B. (1984). Local hyperthermia with interstitial techniques. Cancer Res.

[26] Atkinson W.J., Brezovich I.A., Chakraborty D.P. (1984). Useable frequencies in hyperthermia with thermal seeds. IEEE (Inst. Electr. Electron Eng.) Trans. Biomed. Eng..

[27] Etheridge M.L., Bischof J.C. (2012). Optimizing Magnetic Nanoparticle Based Thermal Therapies within the Physical Limits of Heating. Ann. Biomed. Eng..

[28] Wust P., Nadobny J., Szimtenings M., Stetter E., Gellermann J. (2007). IMPLICATIONS OF CLINICAL RF HYPERTHERMIA ON PROTECTION LIMITS IN THE RF RANGE. Health Phys..

[29] Stigliano R.V., Shubitidze F., Petryk J.D., Shoshiashvili L., Petryk A.A., Hoopes P.J. (2016). Mitigation of eddy current heating during magnetic nanoparticle hyperthermia therapy. Int. J. Hyperth..

[30] Hergt R., Dutz S. (2007). Magnetic particle hyperthermia—Biophysical limitations of a visionary tumour therapy. J. Magn. Magn. Mater..

[31] Shubitidze F., Kekalo K., Stigliano R., Baker I. (2015). Magnetic nanoparticles with high specific absorp-tion rate of electromagnetic energy at low field strength for hyperthermia therapy. J. Appl. Phys..

[32] Bakker F., Paulides M.M., Neufeld E., Christ A., Kuster N., van Rhoon G.C. (2011). Children and adults exposed to electromag-netic fields at the ICNIRP reference levels: Theoretical assessment of the induced peak temperature increase. Phys. Med. Biol..

[33] Christ A., Kainz W., Hahn E.G., Honegger K., Zefferer M., Neufeld E., Rascher W., Janka R., Bautz W., Chen J. (2009). The Virtual Family—Development of surface-based anatomical models of two adults and two children for dosimetric simulations. Phys. Med. Biol..

[34] https://itis.swiss/virtual-population/tissue-properties/database/.

[35] Kekalo K., Baker I. (2013). Magnetic Nanoparticles, Composites, Suspensions and Collids with High Specific Absorption Rate (SAR). Patent.

[36] Kekalo K., Baker I., Meyers R., Shyong J. (2015). Magnetic Nanoparticles with High Specific Absorption Rate at Low Alternating Magnetic Field. Nano LIFE.

[37] Griffith J.M., Pan G.W. (2011). Time Harmonic Fields Produced by Circular Current Loops. IEEE Trans. Magn..

[38] Attaluri A., Jackowski J., Sharma A., Kandala S.K., Nemkov V., Yakey C., Deweese T.L., Kumar A., Goldstein R.C., Ivkov R. (2020). Design and construction of a Maxwell-type induction coil for magnetic nanoparticle hyperthermia. Int. J. Hyperth..

[39] Gneveckow U., Jordan A., Scholz R., Brüß V., Waldöfner N., Ricke J., Feussner A., Hildebrandt B., Rau B., Wust P. (2004). Description and charac-terization of the novel hyperthermia- and thermoablation-systemMFHVR300F for clinical magnetic fluid hyperthermia. Med. Phys..

[40] Pennes H.H. (1948). Analysis of tissue and arterial blood temperatures in the resting human forearm. J. Appl. Physiol..

[41] Razmadze A., Shoshiashvili L., Kakulia D., Zaridze R., Bit-Babik G., Faraone A. (2009). Influence of Specific Absorption Rate Averaging Schemes on Correlation between Mass-Averaged Specific Absorption Rate and Temperature Rise. Electromagnetics.

[42] Ga¨hwiler B., Mamoon A., Schlapfer W., Tobias C. (1972). Effects of temperature on spontaneous bioelectric activity of cultured nerve cells. Brain Res..

[43] Emcos Studio 2022. https://www.emcos.com/.

[44] Prijic S., Sersa G. (2011). Magnetic nanoparticles as targeted delivery systems in oncology. Radiol. Oncol..

